# Characterization of the Channel Constriction Allowing the Access of the Substrate to the Active Site of Yeast Oxidosqualene Cyclase

**DOI:** 10.1371/journal.pone.0022134

**Published:** 2011-07-21

**Authors:** Simonetta Oliaro-Bosso, Giulia Caron, Silvia Taramino, Giuseppe Ermondi, Franca Viola, Gianni Balliano

**Affiliations:** Dipartimento di Scienza e Tecnologia del Farmaco, Facoltà di Farmacia, Università degli Studi di Torino, Turin, Italy; National Institute for Medical Research, Medical Research Council, United Kingdom

## Abstract

In oxidosqualene cyclases (OSCs), an enzyme which has been extensively studied as a target for hypocholesterolemic or antifungal drugs, a lipophilic channel connects the surface of the protein with the active site cavity. Active site and channel are separated by a narrow constriction operating as a mobile gate for the substrate passage. In *Saccharomyces cerevisiae* OSC, two aminoacidic residues of the channel/constriction apparatus, Ala525 and Glu526, were previously showed as critical for maintaining the enzyme functionality. In this work sixteen novel mutants, each bearing a substitution at or around the channel constrictions, were tested for their enzymatic activity. Modelling studies showed that the most functionality-lowering substitutions deeply alter the H-bond network involving the channel/constriction apparatus. A rotation of Tyr239 is proposed as part of the mechanism permitting the access of the substrate to the active site. The inhibition of OSC by squalene was used as a tool for understanding whether the residues under study are involved in a pre-catalytic selection and docking of the substrate oxidosqualene.

## Introduction

In sterol biosynthesis, oxidosqualene cyclases (OSCs) catalyze the most outstanding structural alteration step of the pathway: the shaping of the totally open triterpene intermediate 2,3-oxidosqualene, generated by the mevalonate pathway, into a polycyclic steroid, namely lanosterol in non-photosynthetic eukaryotes or cycloartenol in photosynthetic eukaryotes. In prokaryotes, an enzyme similar to oxidosqualene cyclases, squalene-hopene cyclase (SHC), catalyzes the cyclization of the triterpene squalene. For more than fifty years, a large number of studies has been addressed to the understanding of the complex catalytic mechanism of squalene- and oxidosqualene cyclizing enzymes [1], [2]. Milestones of this long scientific pathway are the solution of the crystal structure of SHC from *Alicyclobacillus acidocaldarius* in 1997 [3] and human OSC (*Hsa*OSC) in 2004 [4]. The structural analyses of both SHC and *Hsa*OSC revealed the essential features of the active sites, and evidenced also the presence of a lipophilic channel connecting the surface of the protein with the active site cavity. Active site and channel are separated by a narrow constriction formed by few aminoacid residues operating as a mobile gate for the substrate passage [5], [6]. The structural analysis of both the channel and the channel constriction of SHC and OSCs suggests that they play a role in capturing the substrate and docking it to the active site cavity. In eukaryotes, the channel might even play a more important role as it could guide the asymmetric substrate squalene epoxide to the active site with the correct orientation, so that the epoxide group interacts with the catalytic residues to trigger the cyclization process [7]. We have previously pointed out the critical role of the channel and the constriction of yeast OSC through an inhibition and site-directed mutagenesis approach [6]. Our results show that two residues, Ala525 and Glu526, close to the channel constriction, are critical for both the protein conformational stability and the susceptibility to irreversible inhibition. In the present study, 16 novel mutants of *S. cerevisiae* (*Sce*OSC) have been prepared and tested for their catalytic activity. Some of them were designed to extend the study of the previously selected position Ala525, others were suggested by the interactions shown in the modelling study by the residues His193, Asn211, His291 [6], others, finally, were designed on the basis of the structural role assigned to the corresponding residues Tyr237 and Cys233 in *Hsa*OSC [4]. To make it possible to compare results of the present and previous study, all the novel mutants have been built starting from the mutant C457D, housing a C→D mutation in the active site cavity, used in our first site-directed mutagenesis study [6].

Several of the novel mutants of this study were less active than the control, pointing out the critical pre-catalytic role of the channel and channel constriction in *Sce*OSC. One of the new mutants studied, C457D/N211K, revealed a high thermal instability, confirming that the alteration of the complex H-bonding network around the Glu526 residue, as previously shown [6], is critical for the enzyme functionality.

Results of the enzymatic assays are discussed with the help of models of the 3D structures of mutant proteins.

We have also checked the ability of the channel and constriction of OSC to properly dock the substrate, and studied the putative different ability of mutants to distinguish epoxide- from non-epoxide end of the substrate by testing their sensitivity to squalene as a possible competitive inhibitor.

The study will contribute to depict structural features that made OSCs capable of a pre-catalitically recognition of the substrate oxidosqualene, a supposed critical step to boost the catalytic reaction. Moreover, the design of new OSC inhibitors, as potential tools for cholesterol-lowering or antifungal/antiparasitic therapy [8], [9], could take advantage from the identification of structurally crucial aminoacid residues.

## Results and Discussion

### *Sce*OSC and mutant models

Firstly, by using the X-Ray structure of the *Hsa*OSC (pdb code: 1W6K), a homology model of *Sce*OSC was built (see Materials and Methods and Table S1 in supporting informations for details).

**Figure 1:**
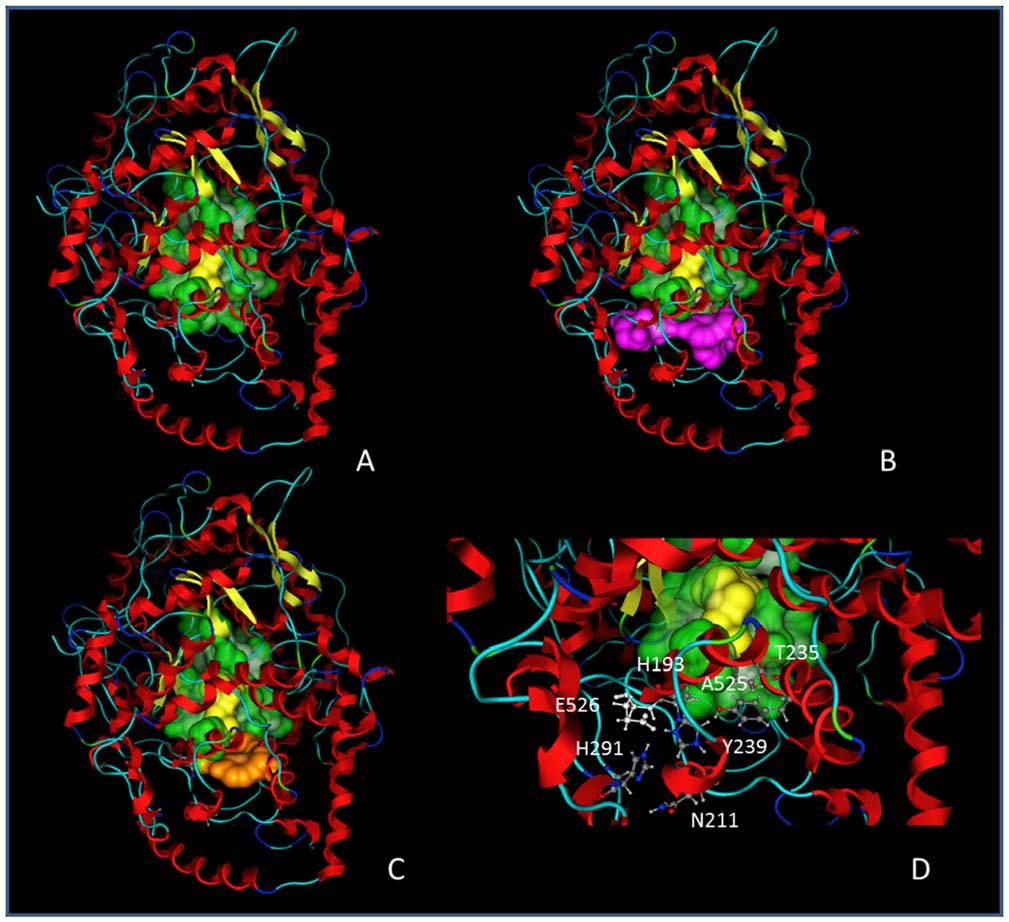
*Sce*OSC and mutant models. A) *Sce*OSC active site (in green) and lanosterol location (in yellow) as deduced from *Hsa*OSC crystallographic data (1W6K). B) Aas involved in the channel constriction (in pink) as suggested by SHC-based considerations. C) Aas involved in the channel constriction (in orange) as suggested *Hsa*OSC-based considerations. D) Mutated residues studied in this work (together with Glu526 shown for comparison).

Once obtained the *Sce*OSC model, its active site (in green in Figure 1A) was recognized by comparison with the *Hsa*OSC structure 1W6K to facilitate the visual detection of the information, to date available, about the channel and its constriction. In particular, Figure 1B shows the four aminoacids (Trp231, Tyr239, Ala525, Glu526) hypothesized to form the channel constriction on the basis of SHC-based considerations, whereas Figure 1C shows the three aminoacids (Tyr239, Thr235 and Ile531) suggested by the literature as responsible for the substrate passage in the *Hsa*OSC structure [4].

The six aminoacid residues (His193, Asn211, His291, Tyr239, Thr235 and Ala525) object of this study, together with Glu526 investigated in a previous report [6], are in Figure 1D which also outlines their relevance to the active site.

Finally, the 3D structures of the 16 novel mutants were obtained by MOE Rotamer Explorer tool as described in Materials and Methods (data not shown).

### Characterization of *Sce*OSC mutants

All the novel mutants are double mutants constructed from the C457D mutant used in our first site-directed mutagenesis study [6]. This mutant, which possesses a C→D mutation in the active site cavity, had initially been constructed as a control to test the sensitivity to thiol reagents of mutants bearing an X→C substitution around the channel constriction [6].

In the present study, the C457D mutant was maintained as a control to make it possible both to compare results of the present and previous study and to compare the sensitivity to thiol reagents of Cys-bearing and non bearing mutants included in the present study.

All the mutated genes were integrated in a SMY8 strain and were able to abolish the auxotrophy for ergosterol of this strain.

Sequence analysis of the 16 mutated *erg7* genes introduced in the OSC-defective SMY8 strain confirmed the presence and the location of the mutation. The catalytic activity of the corresponding proteins was studied in the cell homogenates, by determining the specific activities (Table 1).

**Table 1:** OSC specific activity of the homogenates of the differently transformed SMY8 strains.

*Sce*OSC mutant	* Specific Activity (nmol/h/mg protein)
C457D	1.91±0.095
C457D/A525F	0.14±0.036
C457D/A525Y	0.29±0.090
C457D/A525I	0.73±0.140
C457D/A525L	0.75±0.110
C457D/H193A	0.64±0.058
C457D/H193N	0.49±0.077
C457D/N211A	0.65±0.130
C457D/N211D	0.79±0.150
C457D/N211Q	1.00±0.180
C457D/N211K	0.39±0.073
C457D/H291A	0.64±0.140
C457D/H291N	0.80±0.260
C457D/T235A	0.67±0.130
C457D/T235C	0.85±0.130
C457D/Y239A	0.97±0.130
C457D/Y239F	0.34±0.044

*Values ± SE are the means of duplicate assays of at least two different experiments.

The specific activity of almost all the new mutants was reduced by more than 50% with respect to the control mutant C457D, confirming the critical role of the aminoacid residues under study. As a further control, we prepared, using exactly the same procedure, a **C457D/C619A** mutant, where the mutated residue Cys619 is, according to the structural model, far from both the active site and the access channel. The specific activity of this mutant is 2.3±0.49, not significantly different from that of control mutant C457D.

**Figure 2:**
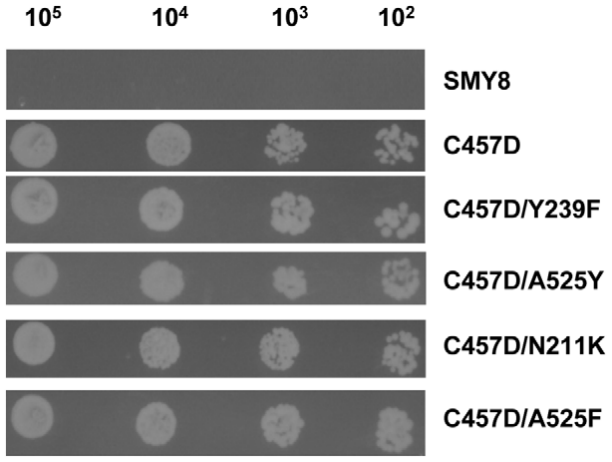
Spot plate analysis of *Sce*OSC mutant strains C457D, C457D/Y239F, C457D/A525Y, C457D/N211K and C457D/A525F. Strains were grown on SC-L media containing galactose supplemented with hemin. Serial tenfold dilutions, giving concentration in the range 10^5^–10^2^ cells, were spotted. Pictures were taken after 4 day incubation.

Four mutants, namely **C457D/A525F**, **C457D/A525Y**, **C457D/Y239F** and **C457D/N211K**, have a very low specific activity, ranging from 10 to 20% of the control C457D. Nevertheless, the low OSC activity of these four mutants was sufficient to support growth in the absence of added ergosterol, as shown by spot plate tests (Figure 2). Under the same conditions, the growth of the defective strain SMY8 was completely abolished.

### A complex network of hydrogen bonds helps in maintaining the conformation of the constriction loop and the full activity of the enzyme

The aminoacid residues in positions 525, 193, 211, 291, according to our model of *Sce*OSC and to the crystal structure of *Hsa*OSC contribute to the stabilization of the channel and channel constriction, forming a wide net of hydrogen bonds. In particular, A525, which is located inward from the channel constriction towards the active site, seems to stabilize the conformation of the constriction loop through a direct interaction of its backbone NH group with the side chain of the in-sequence-neighbouring Asn523, while His193, Asn211, and His291 interact one another and with Glu526, a residue identified in previous work as crucial in keeping the functionality and stability of the yeast enzyme [6].

**Figure 3:**
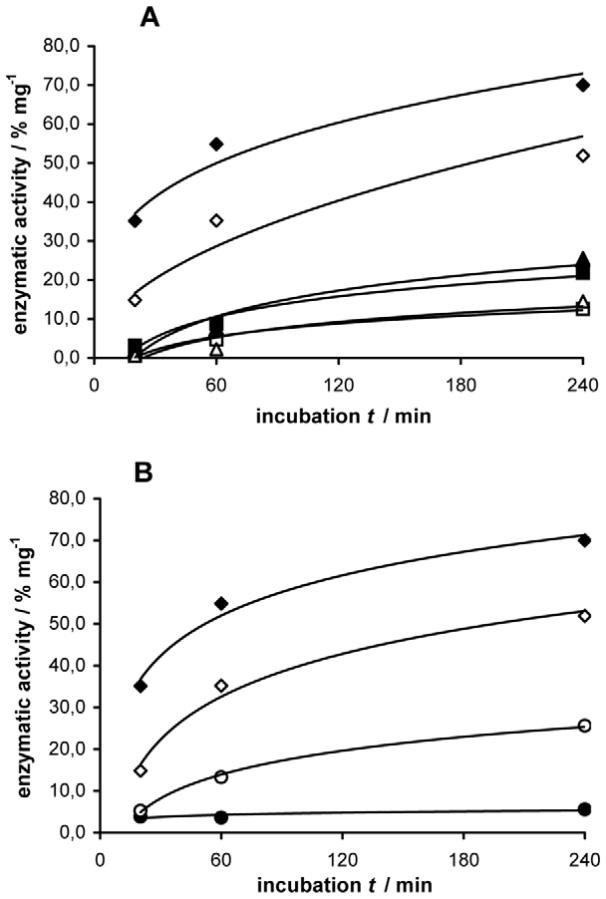
Effect of temperature on the enzymatic activity of mutants C457D, C457D/A525F, C457D/Y239F and C457D/N211K. A) Percentage of labeled product per mg of protein produced by homogenates of mutant C457D (♦,⋄), C457D/A525F (▪,□) and C457D/Y239F (▴,▵) at 35°C (filled symbols) and 25°C (empty symbols), respectively. B) Percentage of labeled product per mg of protein produced by homogenates of mutant C457D (♦,⋄) and C457D/N211K (•, ○) at 35°C (filled symbols) and 25°C (empty labels). Values are the means of at list two separate experiments, each one carried out in duplicate.

**Figure 4:**
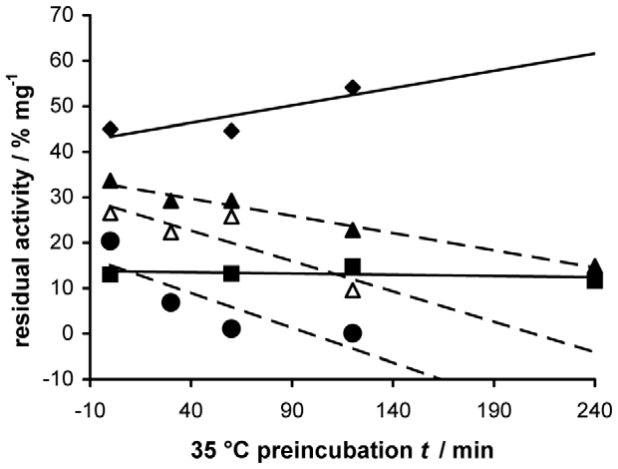
Residual activity of mutants C457D, C457D/A525F, C457D/N211D, C457D/N211A and C457D/N211K, after preincubation at 35°C. Residual enzymatic activity per mg of protein of mutants C457D (♦), C457D/A525F (▪), C457D/N211D (▴), C457D/N211A (▵) and C457D/N211K (•) was determined after 30, 60, 120 and 240 min of preincubation at 35°C in the absence of substrate. Dotted lines are used for the mutants at position 211. After preincubation, the homogenates were incubated with substrate for 4 h at 25°C. Values are the means of at list two separate experiments, each one carried out in duplicate.

The results obtained with the mutants **C457D/A525I**, **C457D/A525L**, **C457D/A525F**, **C457D/A525Y**, show a reduction of activity following the substitution of Ala525 with residues bearing bulky aromatic or alkyl side chains in a bulk-dependent manner: the two mutants A525Y and A525F, bearing aromatic residues, were both less active than the two mutants A525I and A525L, bearing long alkyl side chains (Table 1). In agreement with the enzymatic activity results, the modelling studies show that the Phe or Tyr aromatic side chain fill the channel to the active site much more than the Ile or Leu side chains (see Figure S1 in supporting informations). In addition, we had previously observed that the substitution of Ala525 with a cysteine, a residue with a roughly comparable size, that does not impair the hydrogen bond network, caused only a slight decrease in enzymatic activity [6]. Similarly all the mutants at the position 193, 211, 291, i.e. **C457D/H193A**, **C457D/H193N**, **C457D/N211A**, **C457D/N211D**, **C457D/N211Q**, **C457D/N211K**, **C457D/H291A**, **C457D/H291N**, caused the lowering by at least 50% of the enzymatic activity, the more effective one being the **C457D/N211K**, which in addition proved to be extremely unstable at 35°C, as demonstrated by results of two different experiments of termal inactivation showed in figures 3 and 4. The time-course experiments (Figure 3B) showed that the activity at 35°C of mutant C457D/N211K was close to zero after 20 min and did not increase during 4 h of incubation, but it slowly increased during 4 h of incubation at 25°C, while the activity of control C457D and of the other mutants tested (C457D/A525F and C457D/Y239F) increased regularly at both temperatures. The activity of all the mutants in position 211, determined at 25°C, significantly decreased after preincubation at 35°C in the absence of substrate. The activities of the control C457D and the mutant C457D/A525F were not affected by the preincubation at 35°C, as shown in Figure 4, thus pointing out that mutations different from those at position 211 did not affect the thermal stability of the protein.

This picture is supported by the results of a normal mode analysis (NMA) of *SceOSC* based on an anisotropic netwok model (ANM) [10]. The NMA approach can be used to describe intrinsic protein fluctuations in place of the more computational time demanding Molecular Dynamics. In particular, NMA provided the correlation between the residues fluctuations responsible of the intrinsic motions of the protein around its native state conformation. NMA showed a correlation between the motion of residues in position 193, 211, 291, 525 and 526 (see Materials and Methods, Supporting Text S1, Figure S2 and Table S2 in supporting informations for details). These results reinforce the hypothesis that a modification in the hydrogen bond pattern of any of the five aminoacids (His193, Asn211, His291, Ala525 and Glu526) is expected to alter the whole hydrogen bond pattern of the involved substructure.

In summary, all the substitutions at the position 525 and at the three positions interacting with Glu526, i.e. 193, 211, 291, caused the lowering of the enzymatic activity, the more effective ones being those at position 211, in particular **C457D/N211K**, where the insertion of a Lys residue (in substitution of Asn) between two His residues (193 and 291) possibly positively charged at physiological pH, likely causes an excess of positive charges near the constriction which could be responsible for the reduced activity and thermal instability.

### A change of conformation of a Tyr residue allows the access to the active site through the channel

Substitutions at positions 239 (Tyr) and 235 (Thr) (mutants **C457D/Y239F**, **C457D/Y239A**, **C457D/T235A**, **C457D/T235C**) were designed on the basis of the structural role assigned to the corresponding residues Tyr237 and Cys233 in *Hsa*OSC, that were supposed to change their side chain conformation to allow the passage of the substrate through the channel constriction. Interestingly, the simple removal of an hydroxyl group, as in the C457D/Y239F mutant, caused the most dramatic lowering of the enzyme activity (less than 20% of the control), while the substitution Tyr to Ala only reduced the activity to a half (Table 1).

**Figure 5:**
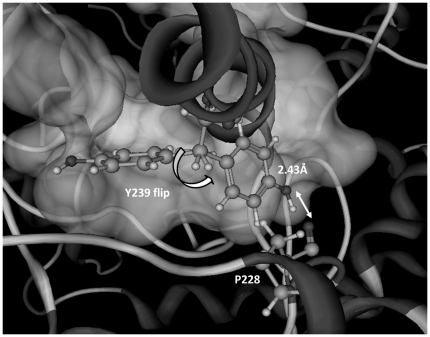
Tyr239 flip. The hydrogen bond between the conformation of Tyr239 enabling the access to the active site and Pro228 is shown (see text for details).

Molecular modelling suggests that Tyr239 fills the middle portion of the lipophilic channel constriction with its bulky side chain. A rotation of the side chain stabilized by a hydrogen bond bridge with Pro228 could open the channel and enable the substrate to enter the active site. The replacement of tyrosine with phenylalanine in mutant Y239F keeps the channel in a closed conformation, possibly causing the observed decrease of enzymatic activity. By replacement of tyrosine with a smaller aminoacidic residue, such as alanine, the channel is probably always open and the substrate can freely access to the catalytic site (Figure 5), also when it is in an uncorrect orientation: this could explain the 50% loss of activity. A similar flip of a tyrosine residue was reported for some enzymes belonging to the catechol 1,2-dioxygenase family [11]. The alignment with Blink (http://www.ncbi.nlm.nih.gov/sutils/blink.cgi) show that most yeast and animal OSCs have a Tyr residue at 239 position (*S. cerevisiae* numbering), whereas most bacterial squalene hopene cyclases (SHC), accepting as a substrate the symmetric molecule of squalene, possess a Val or Ile residue at the corresponding constriction-forming position. We speculate that in the OSCs bearing a Tyr at the 239 position, the control mechanism of the selective access of the substrate to the active site could be partially ascribed to the rotation of the Tyr residue.

**Figure 6:**
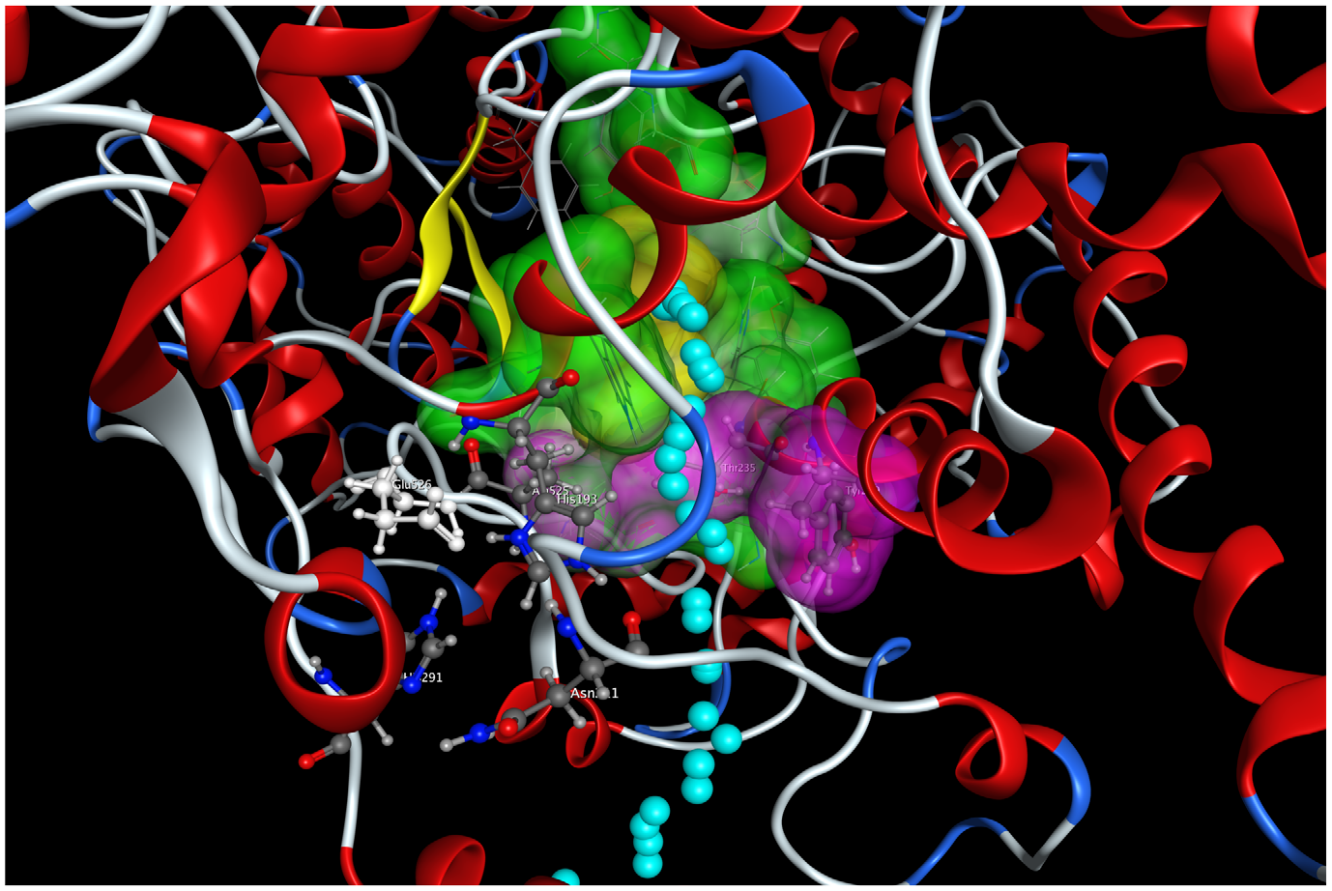
The access route to the active site proposed by CAVER is shown by cyan balls.

As a further support to this hypothesis, by submitting the *Sce*OSC model to CAVER, a program that identifies and visualizes routes from the interior of the protein to the bulk solvent (see Materials and Methods), the access to the active site was found only if the Tyr239 residue is maintained in a rotated position by the formation of a H-bond with Pro228, a condition that cannot be met when the Tyr239 was substituted by a Phe residue (Figure 6).

The mutations **C457D/T235A and C457D/T235C** point out that also the loss of a hydrogen bond between Thr235 and Ile531( (clearly visible in our model of *Sce*OSC), could be relevant for maintaining the conditions for the proper accessibility to the active site. The mutant **C457D/T235C** did not demonstrate a higher sensitivity to the thiol reagent dodecyl-maleimide, showing a minor accessibility than expected.

### Effect of squalene as a competitive inhibitor

The effect of squalene as a possible competitive inhibitor was checked in order to evaluate the role of channel and channel constriction of OSC in the pre-catalytic selection of the substrate. Our hypothesis was that in eukaryotic oxidosqualene cyclases the channel is endowed with an additional function compared to prokaryotic squalene-hopene cyclase (SHC) [12]. It is known that SHC can transform either squalene or oxidosqualene, the latter with a higher efficiency [13], while OSC accepts only oxidosqualene as a substrate. An efficient substrate transformation by OSC could occur only if oxidosqualene settles into the active site with its epoxide properly oriented toward the protonating apparatus responsible for triggering the cyclization process, as it is hard to envisage a flip-flop motion inside the active site [7]. According to this hypothesis, squalene should act as a competitive inhibitor and the channel constriction could operate as an entropic trap able to prefer a properly oriented molecule of substrate, in order to reduce the risk of competitive inhibition. By testing the inhibitory effect of different concentrations of squalene on the control mutant C457D, we observed inhibitory effect only at concentration ≥0.5 mM, a very high concentration, compared with the Km of oxidosqualene which is in the micromolar range [14]. This suggests that the enzyme is not completely indifferent to the competitive action of squalene, though able to overcome its competition with substrate under physiological conditions. We repeated the inhibition test with the homogenates from the 16 mutants, with the working hypothesis that a mutation in a residue involved in substrate discrimination should cause an increased inhibition by squalene. Only in the **N211A** mutant the inhibitory effect of squalene was significantly higher (approx. 30%) than in the control enzyme, pointing out that the Asn211 residue might be one of the structural determinants of the channel involved in the discrimination of a properly oriented substrate. Interestingly, Asn211 is a fully conserved residue throughout OSCs, whereas all the known SHCs possess a Pro (a highly conformation-altering residue) in the corresponding position. The replacement of proline with asparagine could then be an evolution that confers to OSCs the ability of distinguishing squalene from oxidosqualene and properly channelling oxidosqualene.

In conclusion, on the basis of a structural model developed by fitting the sequence of *Sce*OSC to the *Hsa*OSC structure, we studied a series of *Sce*OSC mutants bearing single aminoacid substitutions in positions belonging or neighbouring the channel constriction, which is supposed to operate as a mobile gate for the substrate entering. Our aim was to provide evidences in support of the hypothesis that the channel apparatus plays an essential role in properly docking substrate to the active site. Most substitutions caused a decrease in enzyme activity, thus pointing out the critical role of the channel constriction area for the functionality of the enzyme. The less active mutants were C457D/A525F and C457D/A525Y, confirming the hypothesis of the involment of Ala525 residue in the channel constriction and in a critical hydrogen network. The failure to form a critical hydrogen bond, allowing a conformational change of Tyr239 residue, seems also to be involved in the poor activity of mutant C457D/Y239F, in agreement with the suggestion of Thoma *et al* [4], that a conformational change of Tyr239 is necessary for the entering of the substrate.

Moreover, a deep alteration of hydrogen bond network could explain the poor activity and thermal instability of mutants at the position 211, particularly of C457D/N211K. In addition, experiments with squalene as inhibitor pointed out that the residue Asn211 could be involved in the specific pre-catalytic recognition of the substrate.

**Figure 7:**
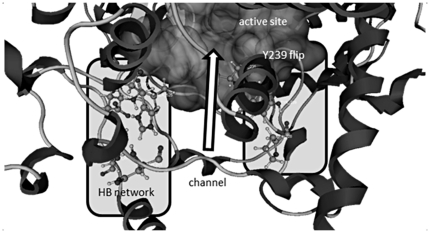
Results summing-up. A complete depiction of the structural requirements of the channel apparatus conferring to OSCs the ability (absent in SHCs) of a pre-catalytical recognition of the substrate is the challenge of the next future. Other mutants shall be built and tested starting from the most highly conserved residues of the SHC channel, replaced by different, yet highly conserved, residues in eukaryotic OSCs.

Figure 7 summarizes in a schematic way all the results found in this study. The integrity of the channel to the active site is guaranteed by a hydrogen bond network formed by His193, Asn211, His291 and Glu526, whereas the active site accessibility is due to the Tyr239 flip.

These results can contribute to design novel site-directed inhibitors of OSCs for a potential use as antifungal or hypocholesterolemic drugs.

## Materials and Methods

### Chemicals

All the components of buffers and cultural media were obtained from Sigma-Aldrich (Milan, Italy) unless otherwise specified. Molecular biology reagents were obtained from Promega Italia unless otherwise specified. Squalene (S) and 2,3-oxidosqualene (OS) were prepared as previously described [15]. The labeled [^14^C]-(3S)2,3-oxidosqualene was obtained through biological synthesis by incubating R,S[2-^14^C]mevalonic acid (1 µCi, 55 mCi/mmol, 2.04 GBq/mmol) (Amersham Pharmacia Biotech, U.K.) with a pig liver S10 supernatant, in the presence of the OSC inhibitor U-14266A [16], as previously described [17].

### Yeast strains and culture conditions

The strain *S. cerevisiae*, lanosterol synthase mutant SMY8 (*MATa erg7::HIS3 hem1::TRP1 ura3-52-trpl-Δ63 leu2-3.112 his3-Δ200 ade2 Gal^+^*) was kindly provided by Professor S.P.T. Matsuda (Department of Chemistry and Biochemistry and Cell Biology, Rice University Houston, Texas-USA) [18].

SMY8 cells were grown to the early stationary phase at 30°C in YPD medium (1% yeast extract, 2% peptone, 2% dextrose) supplemented with hemin (0.013 mg/mL) and ergosterol (0.02 mg/mL). Hemin is needed in the medium as the SMY8 strains contains a mutation (*hem1::TRP1*) affecting the heme biosynthesis. The presence of a heme mutant background is necessary for the viability of lanosterol synthase mutants in aerobic conditions [18]. SMY8 transformants were selected on synthetic complete media plates without leucine, containing yeast nitrogen base (0.67%), dextrose (2%), aminoacids (0.2%), nitrogen base (0.5%), agar (2%) and supplemented with hemin (0.013 mg/mL) and ergosterol (0.02 mg/mL). SMY8 transformed with pSM61.21 plasmids derivatives were analyzed for ergosterol autotrophy by plating on YPG plates (1% yeast extract, 2% peptone, 2% galactose, 2% agar) supplemented with hemin (0.013 mg/mL).

For spot plate analyses, strains were grown on synthetic complete media plates without leucine containing yeast nitrogen base (0.67%), galactose (2%), aminoacids (0.2%), nitrogen base (0.5%) and supplemented with hemin. Each colony was spotted using 5 µl from a 4×105 cells/ml cell suspension. Plates were incubated at 30°C for 96 h and scored.

### Site-directed mutagenesis

The preparation of yeast OSC mutant C457D has been previously described [6].

To prepare the 16 novel double mutants, uracil-containing single-stranded DNAs were prepared from phagemid pSM61.21 C457D in *E. coli* strain RZ1032 coinfected with M13K076 helper phage. Mutagenic oligonucleotide was used to prime the second strand synthesis with T4 polymerase and the DNA circle was closed with T4 ligase [19]. *E. coli* strain DH5α was transformed with the putative mutant construct and clones with the desired mutation were validated by DNA sequencing.

Lithium acetate was used to transform the *S. cerevisiae* lanosterol synthase mutant SMY8 with all the constructs [19] and transformed cells were selected on media plates as described above. Genomic DNA was isolated from yeast using glass beads and phenol [19], and the OSC mutant genes were amplified with PCR and sequenced.

DNA sequence analysis was carried out at the C.R.I.B.I. - BMR Servizio Sequenziamento DNA, Padova (Italy). Alignments were obtained with MultiAlin programme (http://prodes.toulouse.inra.fr/multalin/multalin.html).

### Oxidosqualene cyclase activity

Cell-free homogenates were obtained as previously described [20]. Briefly, after lysis of the cell wall with lyticase the spheroplasts were homogenized with a Potter device. Proteins in the homogenate were quantified with a protein assay kit (Sigma), based on the method of Lowry modified by Peterson [21] and by using bovine serum albumin as a standard. OSC activity was assayed as previously described [22]. Briefly, the homogenates were incubated with the labeled [14C]-(3S)2,3-oxidosqualene (1000 cpm) in MES/TRIS buffer (10 mM), containing EDTA (0.2 mM),Triton X-100 (1 mg/mL of final volume), Tween-80 (0.2 mg/mL of final volume), pH 6.9. The standard incubation time was 30 min at 35°C, but different times and temperatures were used for some experiments as specified in the results. The enzymatic reaction was terminated by the addition of KOH in methanol (10% p/v), the lipids were saponified at 80°C for 30 min, and the nonsaponifiable lipids were extracted with petroleum ether. Extracts were spotted on TLC plates with n-hexane/ethyl acetate (85∶15) as the developing solvent. The conversion of the labeled substrate to labeled product was determined by using a System 200 imaging scanner ( Hewlett Packard, Palo Alto CA, USA).

### Oxidosqualene cyclase inhibition by squalene and dodecyl-maleimide

OSC inhibition was carried out, as described above, by incubating the homogenate with the labeled [^14^C]-(3S)2,3-oxidosqualene (1000 cpm) diluted with cold (R,S)2,3-oxidosqualene to a final concentration 25 µm, in the presence of dodecyl-maleimide (1 mM, 0.2 mM, 25 µM) or squalene (500 µM), respectively for 30 min and 1 h at 35°C. When squalene was the inhibitor, the concentration of Tween-80 and Triton X-100 was respectively 0.1 mg/mL and 1 mg/mL of final volume.

### Computational methods

Molecular modeling studies were performed both on Linux and Windows machines. The following software were used: a) MOE 2008.10 (http://www.chemcomp.com/) for sequence alignment and homology modeling of the *Sce*OSC enzyme wild type and of the mutants; b) ANM (Anisotropic network model) for the analysis of vibrational motions (http://ignmtest.ccbb.pitt.edu/cgi-bin/anm/anm1.cgi); c) CAVER (http://loschmidt.chemi.muni.cz/caver/index.php) for identifying and visualizing routes from the interior of the protein to the bulk solvent and d) Pymol (http://pymol.sourceforge.net/) for visualization and figures preparation.

### Homology modeling

Sequence information of *Sce*OSC was taken from the UniProtKB/Swiss-Prot database (http://www.uniprot.org/uniprot/P38604). The X-Ray structure used as template for homology modeling was downloaded from the Protein Data Bank (*Hsa*OSC, pdb code 1w6k).

Homology modeling was performed by using the homology model tool of MOE. By default, MOE-homology produces 10 models, each is generated by making a series of Boltzmann-weighted choices of side chain rotamers and loop conformations from a set of protein fragments selected from the built-in library of high-resolution protein structures. Starting from the *Sce*OSC sequence (see above) and the *Hsa*OSC template structure (see above), MOE generates ten energy-minimised models (M1-M10) which were checked by both by MOE homology score and WHAT IF (http://swift.cmbi.ru.nl/servers/html/index.html). Both tools individuated one model M8 as the best, and this was submitted to a final minimization using the AMBER force field until the RMS (Root mean Square) gradient was below 0.1 kcal/mol; this was finally used as the *Sce*OSC model.

### Computational introduction of mutations

Mutants were built by applying the MOE tool named Rotamer Explorer on the final homology model described above. For any mutant, all conformers proposed by Rotamer Explorer were investigated but given the limited variations of extracted information, results refer to the conformer with the lowest energy. Briefly, an important component of any modeling method to build mutants is the prediction of side-chain conformations. The Rotamer Library in MOE is a collection of all-atom side-chain conformations derived from high quality X-ray crystal structures. These conformations differ markedly from the traditional rotamer libraries in that bond length and angle variations are permitted. The intent of the MOE rotamer library is to provide adequate coverage of the conformational space available to a sidechain and not just encode dihedral preferences. Given the use of Rotamer Explorer method, no additional minimization run was performed on the obtained mutants.

### Analysis of vibrational motions

ANM (Anisotropic network model) was used to perform the analysis of vibrational motions of *Sce*OSC. Briefly, ANM [23] is a fast computational tool that provides a simulation of spatial fluctuations of proteins in good agreement with traditional CPU time-consuming normal-mode analysis using force fields of molecular dynamics (MD) packages.

The homology model of *Sce*OSC was used as an input to investigate the first 20 dominant modes (good representation of all modes) with standard cutoff values (15 Å).

## Supporting Information

Figure S1 **Mutations on position 525 fill in a different way the channel to the binding site (shown surface).** Left: control mutant C457D; middle: C457D/A525I; right: C457D/A525F.
(TIF)

Figure S2 **The correlations in fluctuations between residues for residues His193 (black circles), Asn211 (grey triangles), His291 (white squares), Glu526 (empty triangle) and Cys457 (grey crosses) as a comparison.**
(TIF)

Text S1 **Detailed results of ANM study.**
(DOC)

Table S1 ***Sce*** **OSC active site involved residues as deduced after superposition with** ***Hsa*** **OSC.**
(DOC)

Table S2 **MOE Protein contacts tool monitors the network of interactions of residues 193, 211 and 291 in investigated mutants.**
(DOC)
