# Supplementary figures and images for: Characterization of the Channel Constriction Allowing the Access of the Substrate to the Active Site of Yeast Oxidosqualene Cyclase
Source: PLoS One. 2011 Jul 21;6(7):e22134. doi: 10.1371/journal.pone.0022134 (PMC3141018; doi:10.1371/journal.pone.0022134)

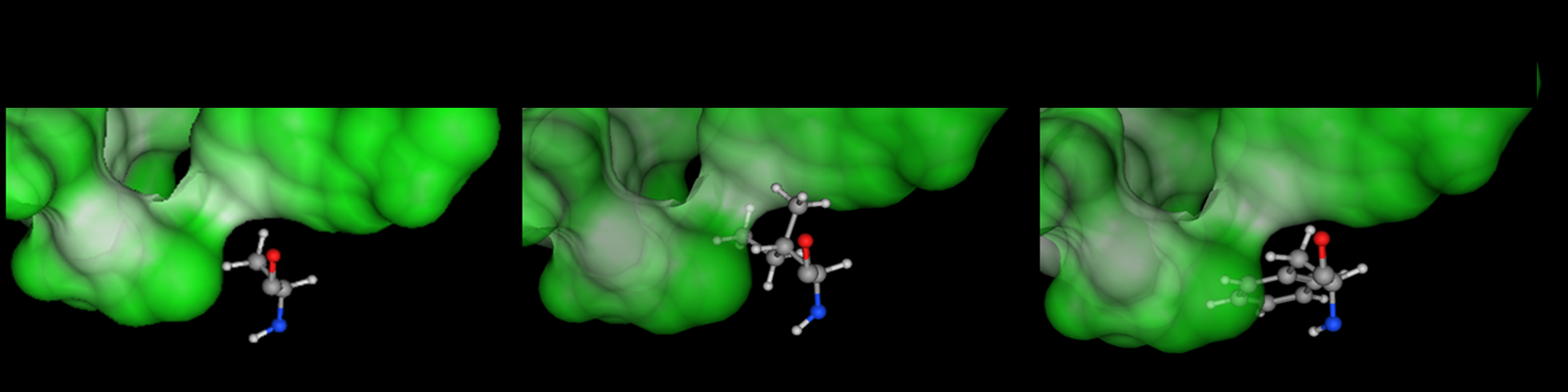

Supplement: Figure S1 — Mutations on position 525 fill in a different way the channel to the binding site (shown surface). Left: control mutant C457D; middle: C457D/A525I; right: C457D/A525F. (TIF) [file pone.0022134.s001.tif]

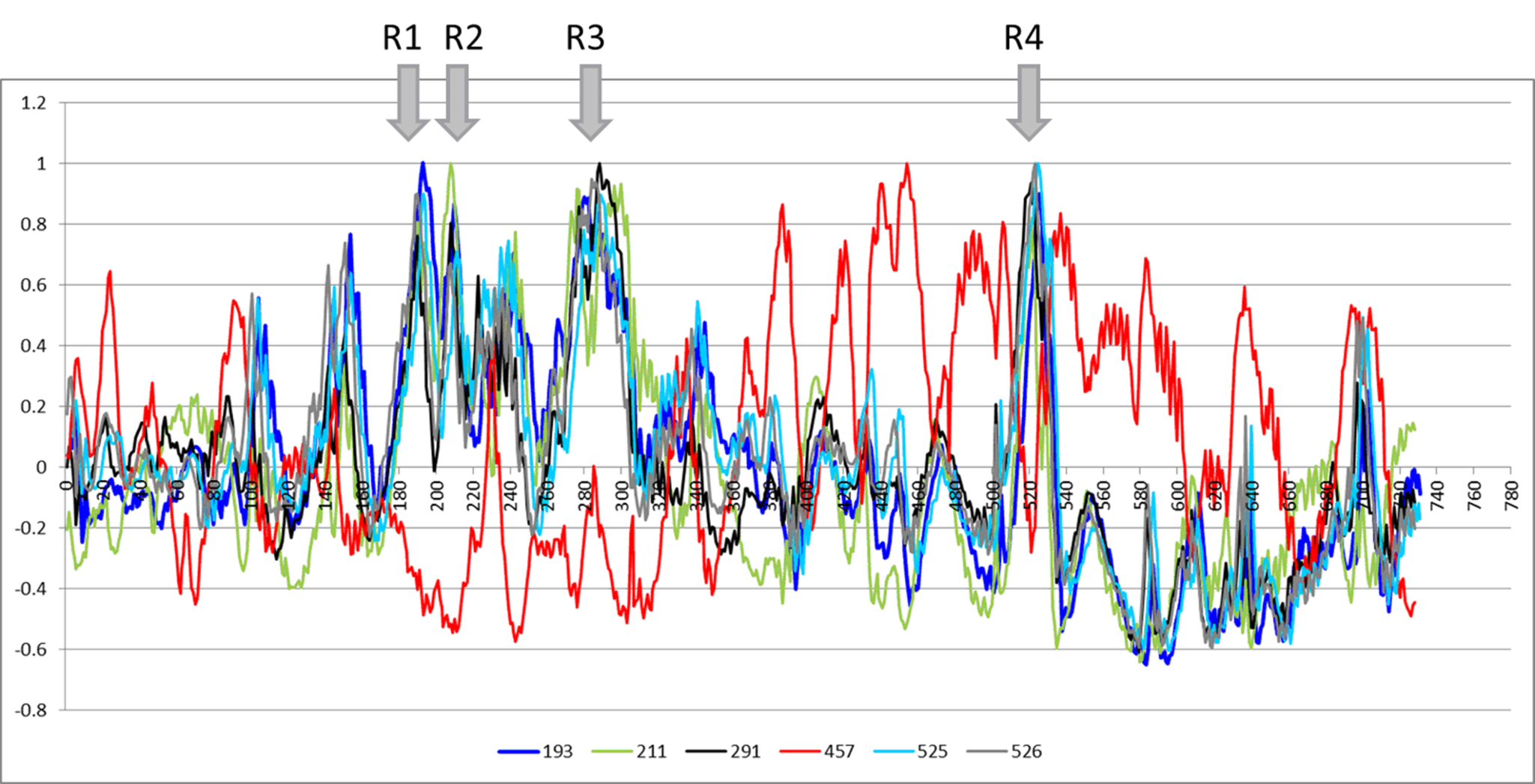

Supplement: Figure S2 — The correlations in fluctuations between residues for residues His193 (black circles), Asn211 (grey triangles), His291 (white squares), Glu526 (empty triangle) and Cys457 (grey crosses) as a comparison. (TIF) [file pone.0022134.s002.tif]
