# Supplementary material for: Characterization of the Channel Constriction Allowing the Access of the Substrate to the Active Site of Yeast Oxidosqualene Cyclase
Source: PLoS One. 2011 Jul 21;6(7):e22134. doi: 10.1371/journal.pone.0022134 (PMC3141018; doi:10.1371/journal.pone.0022134)
Supplement: Text S1 — Detailed results of ANM study. (DOC) [file pone.0022134.s003.doc]

**Detailed results of ANM study**

An anisotropic network model (ANM) study of the wild type SceOSC (see Experimental Section for details) confirmed that residues His193, Asn211, His291, Ala525 and Glu526 interact one another. For example, Figure S1 shows the correlation in fluctuations for residues His193 (blue line), Asn211 (green line), His291 (dark grey line), Ala525 (light blue line), Glu526 (light grey line) and Cys457 (red line) which was chosen as a comparison. Cys457 is located in a different protein region and its motion is securely not correlated with the motion of the five residues cited above. Each point of the graph indicates the cross correlation between the investigated residue and any other residue of the protein. The cross-correlations theoretically vary in the range [-1,+1], the upper and lower limits corresponding to the fully correlated, and fully anticorrelated fluctuations, respectively. Uncorrelated fluctuations, on the other hand, yield 0 as a correlation value.[8] Figure S1 reveals four regions (R1, R2, R3 and R4) in which His193, Asn211, His291, Ala525 and Glu526 are strongly positively cross-correlated one with the others, whereas, as expected, Cys457 has a complete different pattern.

The ANM study thus suggests that a modification in the hydrogen bond pattern of any of the five aminoacids (His193, Asn211, His291, Ala525 and Glu526) is expected to alter the whole hydrogen bond pattern of the involved substructure.
