# Supplementary material for: Characterization of the Channel Constriction Allowing the Access of the Substrate to the Active Site of Yeast Oxidosqualene Cyclase
Source: PLoS One. 2011 Jul 21;6(7):e22134. doi: 10.1371/journal.pone.0022134 (PMC3141018; doi:10.1371/journal.pone.0022134)
Supplement: Table S1 — Sce OSC active site involved residues as deduced after superposition with Hsa OSC. (DOC) [file pone.0022134.s004.doc]

| *Hsa*OSC | *Sce*OSC |
| --- | --- |
| ASP455 | Asp456 |
| TYR98 | Tyr99 |
| TRP192 | Trp194 |
| TRP230 | Trp232 |
| HIS232 | His234 |
| GLY380 | Gly383 |
| TRP387 | Trp390 |
| PHE444 | Phe445 |
| THR502 | Thr509 |
| TYR503 | Tyr510 |
| ILE524 | Ile531 |
| TRP581 | Trp587 |
| PHE696 | Phe699 |
| ASN697 | Asn700 |
| TYR704 | Tyr707 |

**Table S1. *Sce*OSC active site involved residues as deduced after superposition with *Hsa*OSC.**
