# Supplementary material for: Characterization of the Channel Constriction Allowing the Access of the Substrate to the Active Site of Yeast Oxidosqualene Cyclase
Source: PLoS One. 2011 Jul 21;6(7):e22134. doi: 10.1371/journal.pone.0022134 (PMC3141018; doi:10.1371/journal.pone.0022134)
Supplement: Table S2 — MOE Protein contacts tool monitors the network of interactions of residues 193, 211 and 291 in investigated mutants. (DOC) [file pone.0022134.s005.doc]

|  | 193 | 211 | 291 |
| --- | --- | --- | --- |
| Homology model *Sce*OSC | Asn 211 (HB) | His 193 (HB) | Glu 526 (HB) |
|  | Glu 526 (HB) | Asn 280 (HB) |  |
|  | Glu 526 (ion) | Asn 299 (HB) |  |
| C457D-H193A | ---------------  Glu 526 (HB) | ---------------  Asn 280 (HB) | Glu 526 (HB) |
|  | --------------- | Asn 299 (HB) |  |
| C457D-H193N | ---------------  Glu 526 (HB) | ---------------  Asn 280 (HB) | Glu 526 (HB) |
|  | --------------- | Asn 299 (HB) |  |
| C457D-N211A | Ala 211 (HB) | His 193 (HB) | Glu 526 (HB) |
|  | Glu 526 (HB) | --------------- |  |
|  | Glu 526 (ion) | --------------- |  |
| C457D-N211D | Asp 211 (HB) | His 193 (HB) | Glu 526 (HB) |
|  | Glu 526 (HB) | --------------- |  |
|  | Glu 526 (ion) | --------------- |  |
| C457D-N211Q | Gln 211 (HB) | His 193 (HB) | Glu 526 (HB) |
|  | Glu 526 (HB) | --------------- |  |
|  | Glu 526 (ion) | --------------- |  |
| C457D-N211K | Lys 211 (HB) | His 193 (HB) | Glu 526 (HB) |
|  | Glu 526 (HB) | Glu 526 (ion) |  |
|  | Glu 526 (ion) | --------------- |  |
| C457D-H291A | Asn 211 (HB) | His 193 (HB) | --------------- |
|  | Glu 526 (HB) | Asn 280 (HB) | --------------- |
|  | Glu 526 (ion) | Asn 299 (HB) | --------------- |
| C457D-H291N | Asn 211 (HB) | His 193 (HB) | --------------- |
|  | Glu 526 (HB) | Asn 280 (HB) | --------------- |
|  | Glu 526 (ion) | Asn 299 (HB) | --------------- |

**Table S2. MOE Protein contacts tool monitors the network of interactions of residues 193, 211 and 291 in investigated mutants.**
